# Supplementary material for: Shoulder Physiological Offset Parameters in Asian Populations—A Magnetic Resonance Imaging Study
Source: Diagnostics (Basel). 2025 Jan 9;15(2):146. doi: 10.3390/diagnostics15020146 (PMC11763603; doi:10.3390/diagnostics15020146)
Supplement: Supplementary file 1 [file diagnostics-15-00146-s001.zip › Table S2.pdf]

Table S2. Post-hoc power analysis for comparison by grade of osteoarthritis

	HO	GO	LGHO	HAO	CO
Power	0.35	0.293	0.050	0.063	0.055
