# Supplementary material for: Shoulder Physiological Offset Parameters in Asian Populations—A Magnetic Resonance Imaging Study
Source: Diagnostics (Basel). 2025 Jan 9;15(2):146. doi: 10.3390/diagnostics15020146 (PMC11763603; doi:10.3390/diagnostics15020146)
Supplement: Supplementary file 1 [file diagnostics-15-00146-s001.zip › Table S3.pdf]

**Table S3** Intraclass correlation coefficient

| | HO | GO | LGHO | HAO | CO |
|---|---|---|---|---|---|
| Intraobserver ICC | 0.89 [0.81,0.94] | 0.90 [0.82, 0.94] | 0.98 [0.98, 0.99] | 0.87 [0.78, 0.93] | 0.87 [0.78, 0.93] |
| Interobserver ICC | 0.98 [0.97, 0.99] | 0.99 [0.98, 0.99] | 1.00 [0.99, 1.00] | 0.98 [0.96, 0.99] | 0.98 [0.96, 0.99] |

*data is expressed as mean value [95% CI]
