# Supplementary figures and images for: Shoulder Physiological Offset Parameters in Asian Populations—A Magnetic Resonance Imaging Study
Source: Diagnostics (Basel). 2025 Jan 9;15(2):146. doi: 10.3390/diagnostics15020146 (PMC11763603; doi:10.3390/diagnostics15020146)

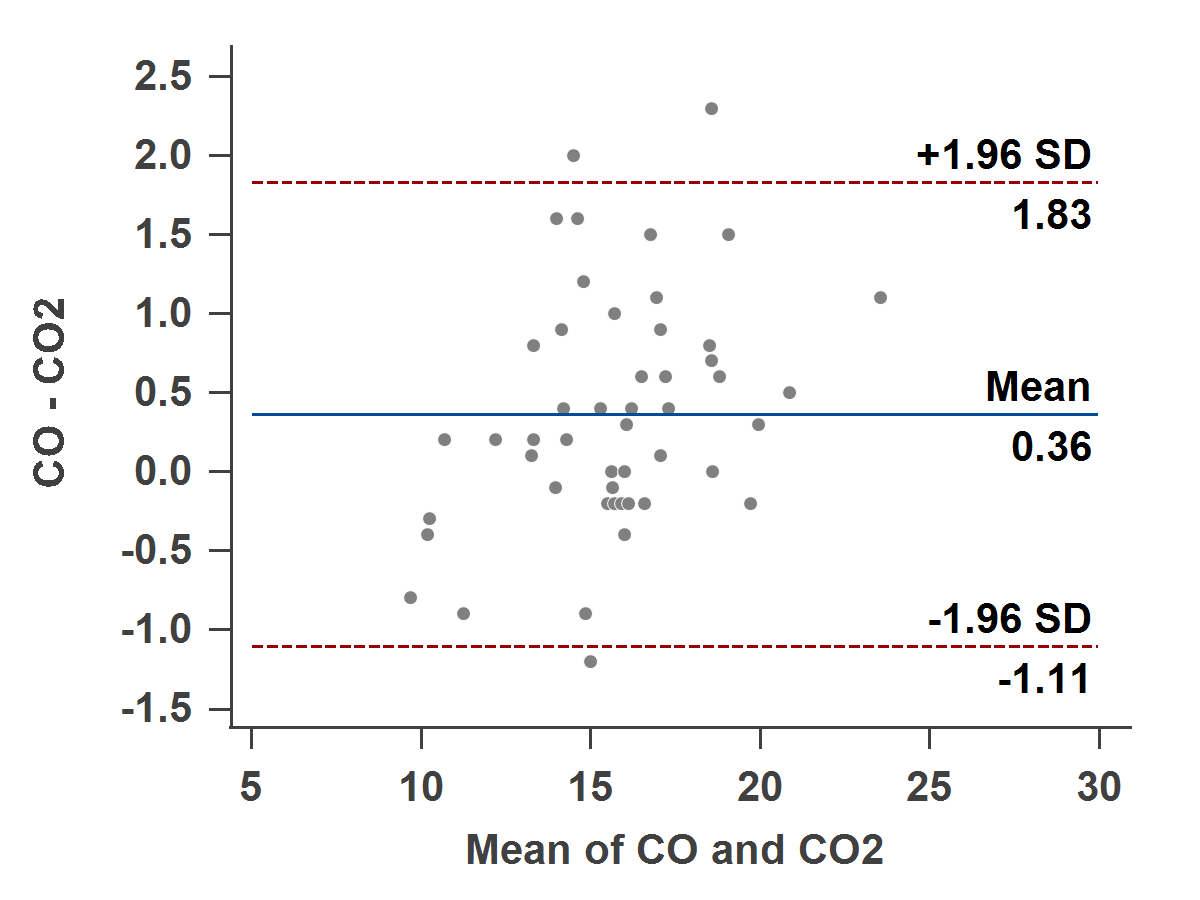

Supplement: Supplementary file 1 [file diagnostics-15-00146-s001.zip › Figure S10_CO.tif]

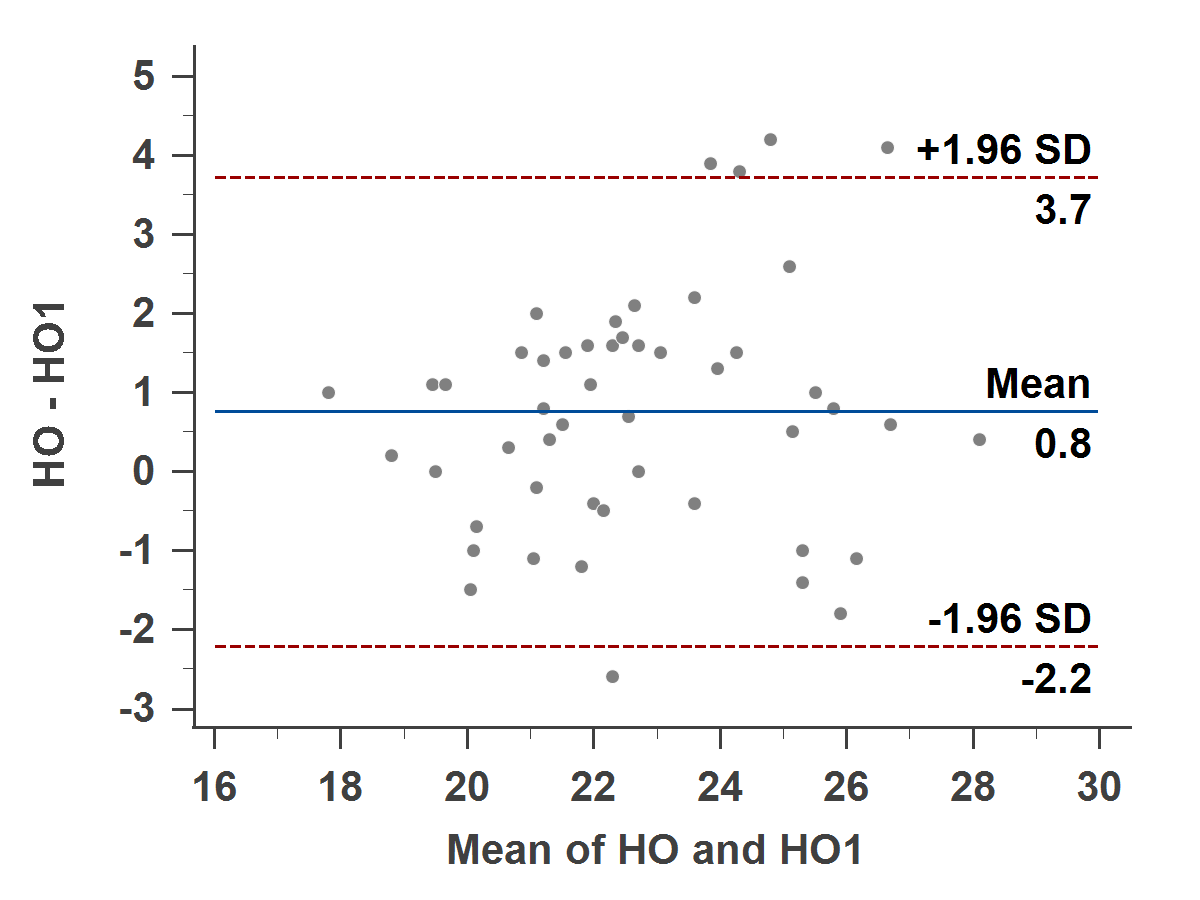

Supplement: Supplementary file 1 [file diagnostics-15-00146-s001.zip › Figure S1_HO.tif]

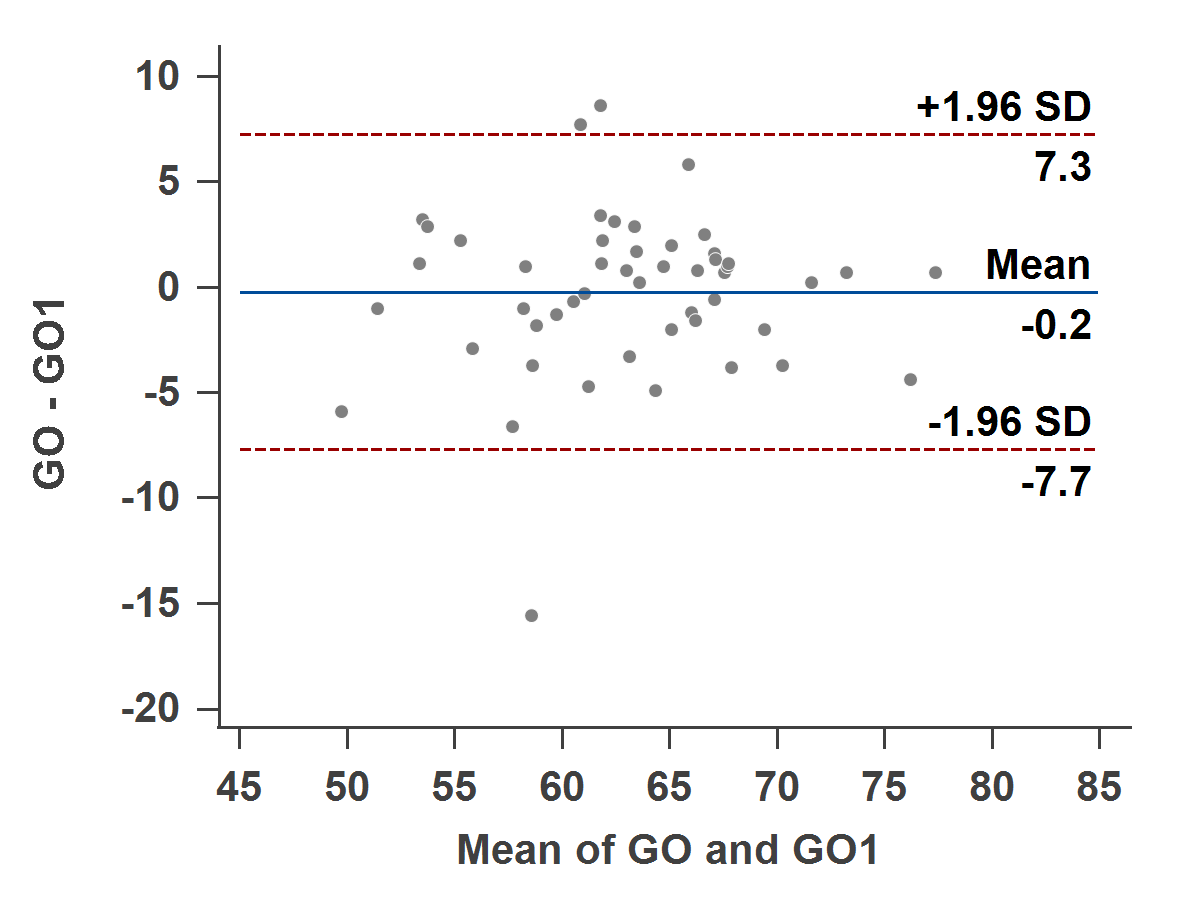

Supplement: Supplementary file 1 [file diagnostics-15-00146-s001.zip › Figure S2_GO.tif]

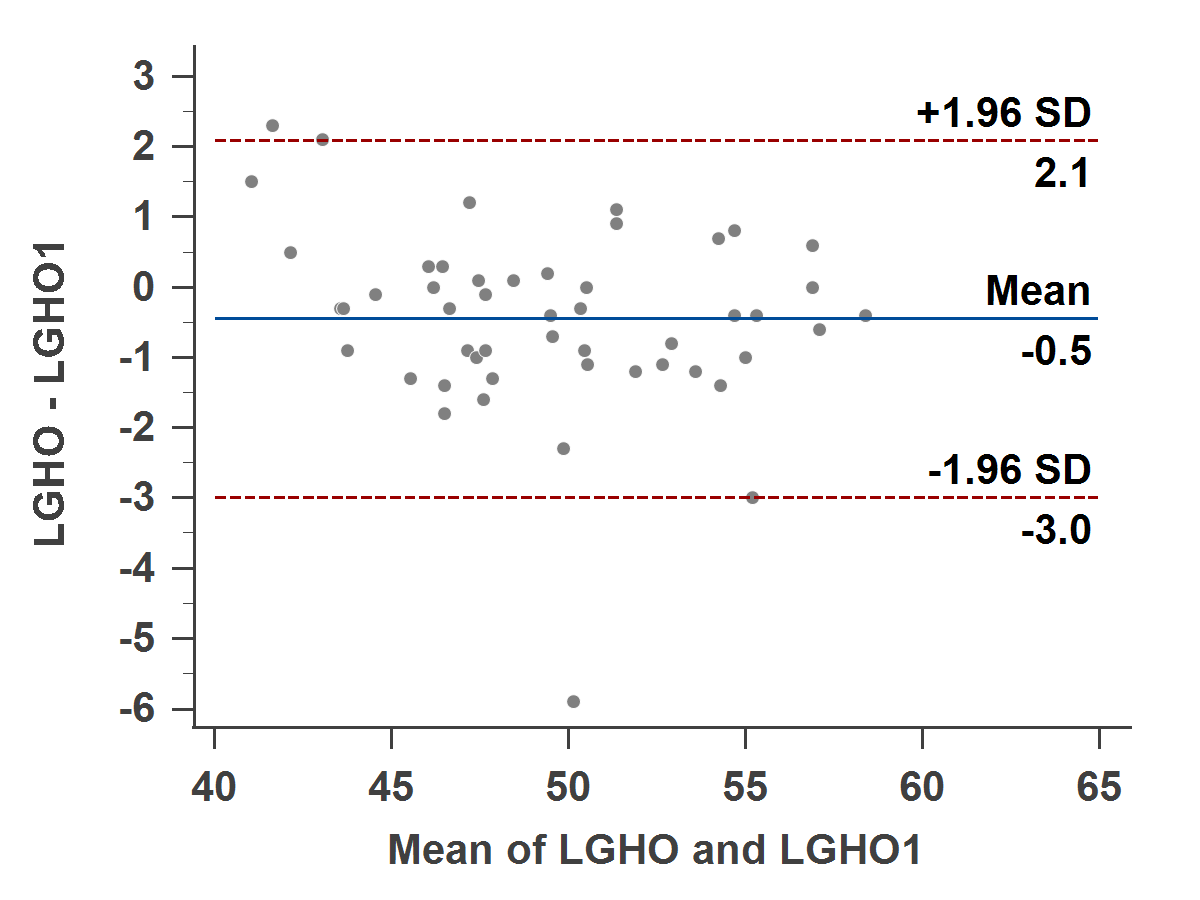

Supplement: Supplementary file 1 [file diagnostics-15-00146-s001.zip › Figure S3_LGHO.tif]

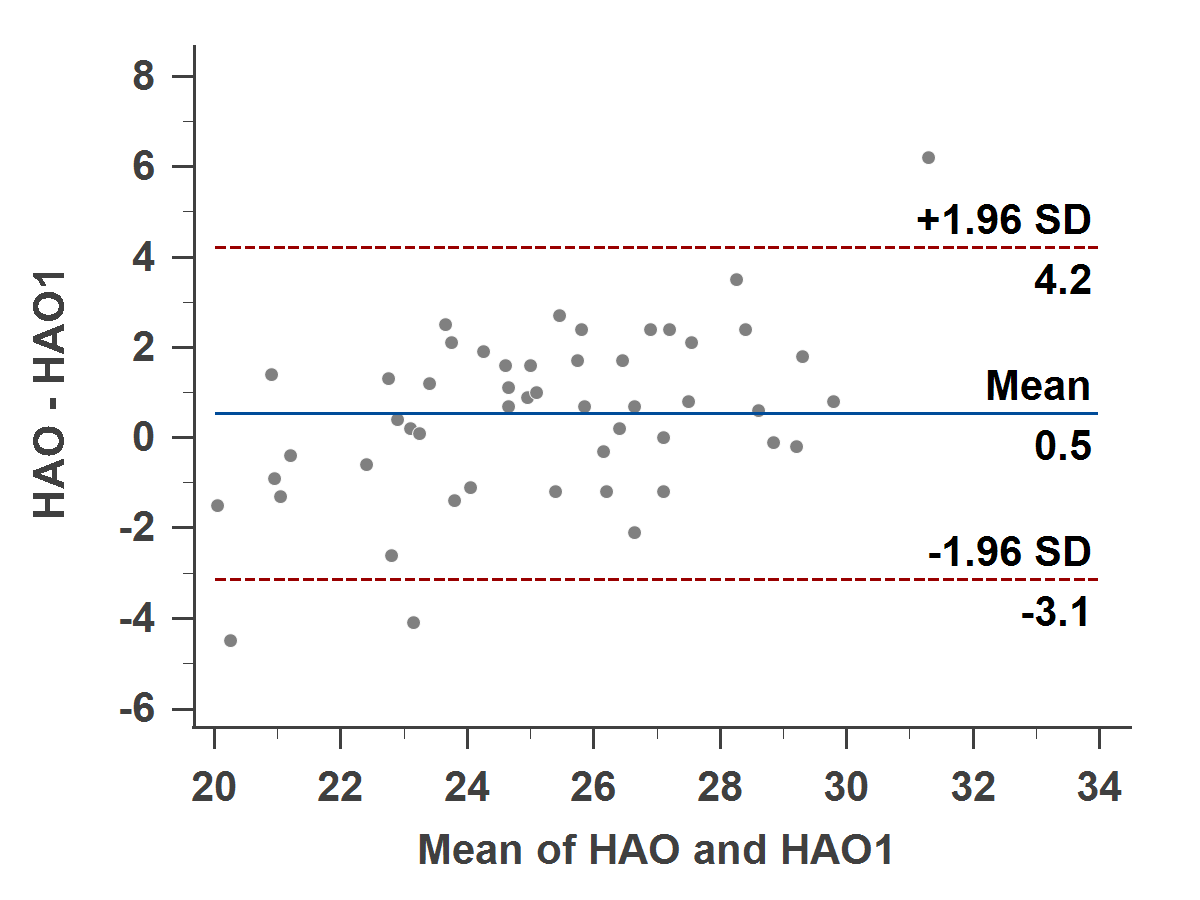

Supplement: Supplementary file 1 [file diagnostics-15-00146-s001.zip › Figure S4_HAO.tif]

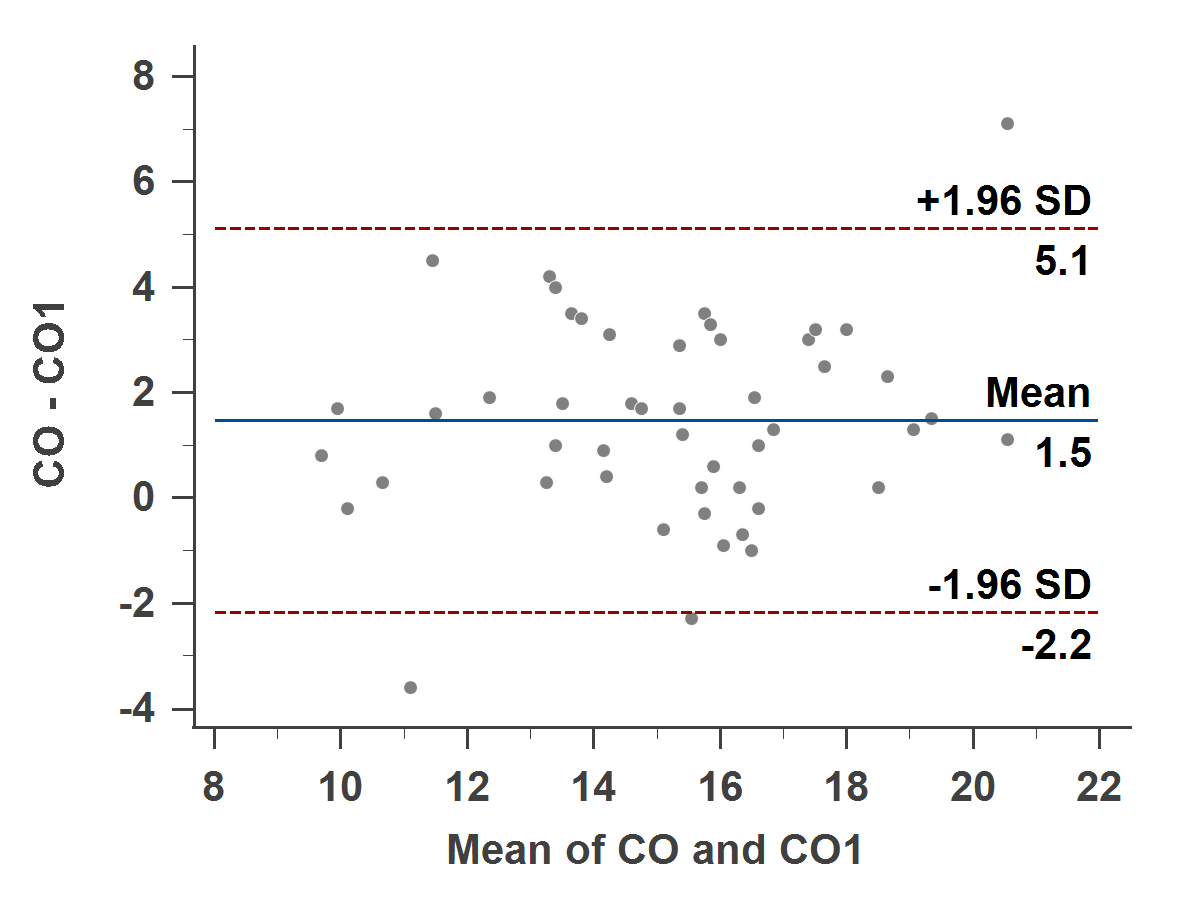

Supplement: Supplementary file 1 [file diagnostics-15-00146-s001.zip › Figure S5_CO.tif]

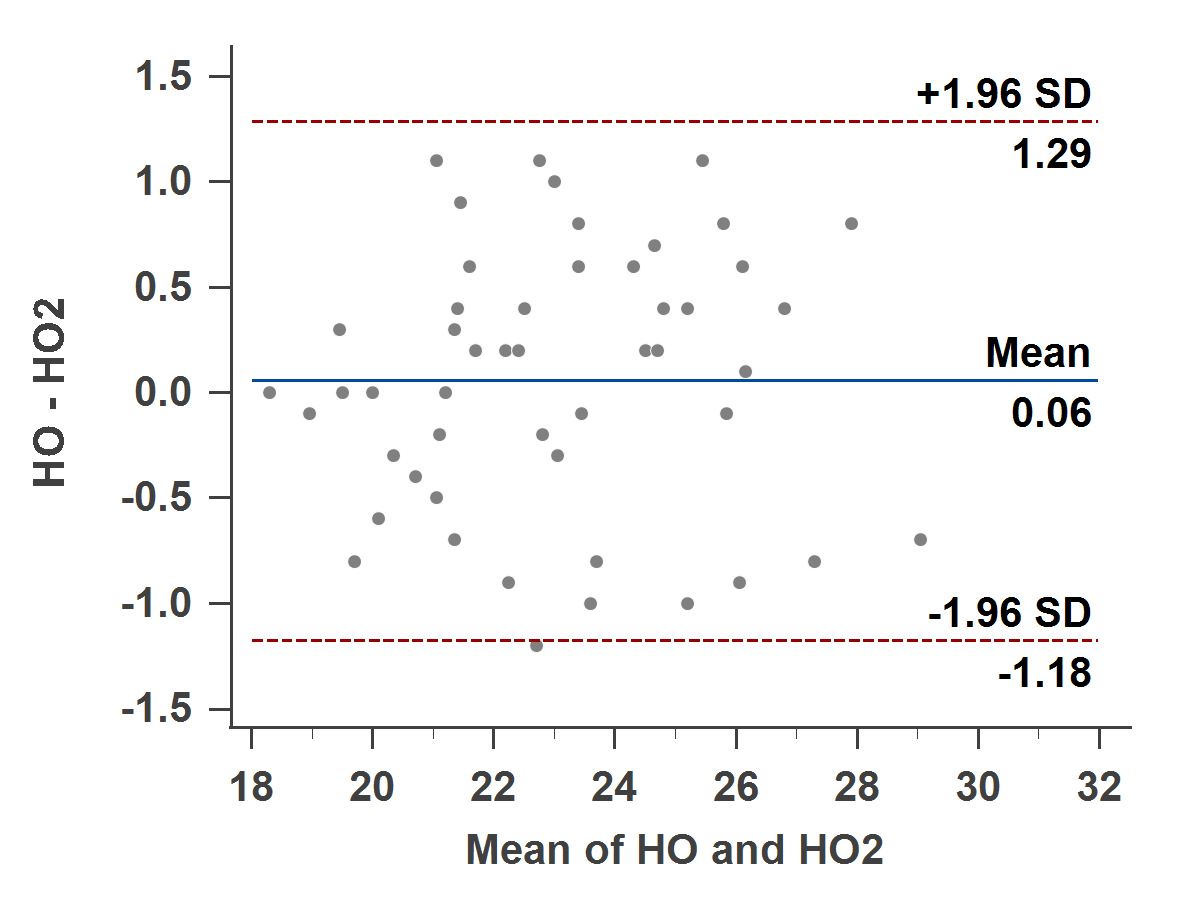

Supplement: Supplementary file 1 [file diagnostics-15-00146-s001.zip › Figure S6_HO.tif]

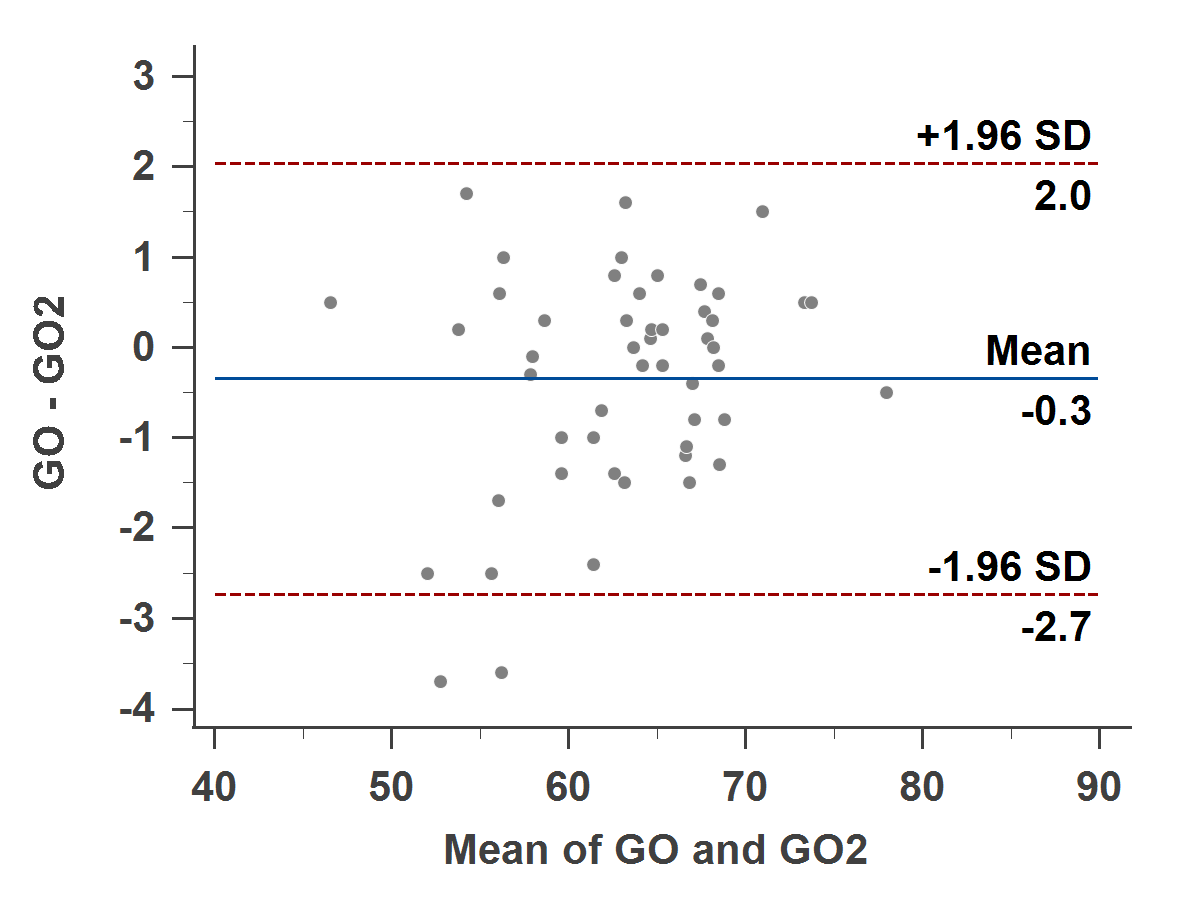

Supplement: Supplementary file 1 [file diagnostics-15-00146-s001.zip › Figure S7_GO.tif]

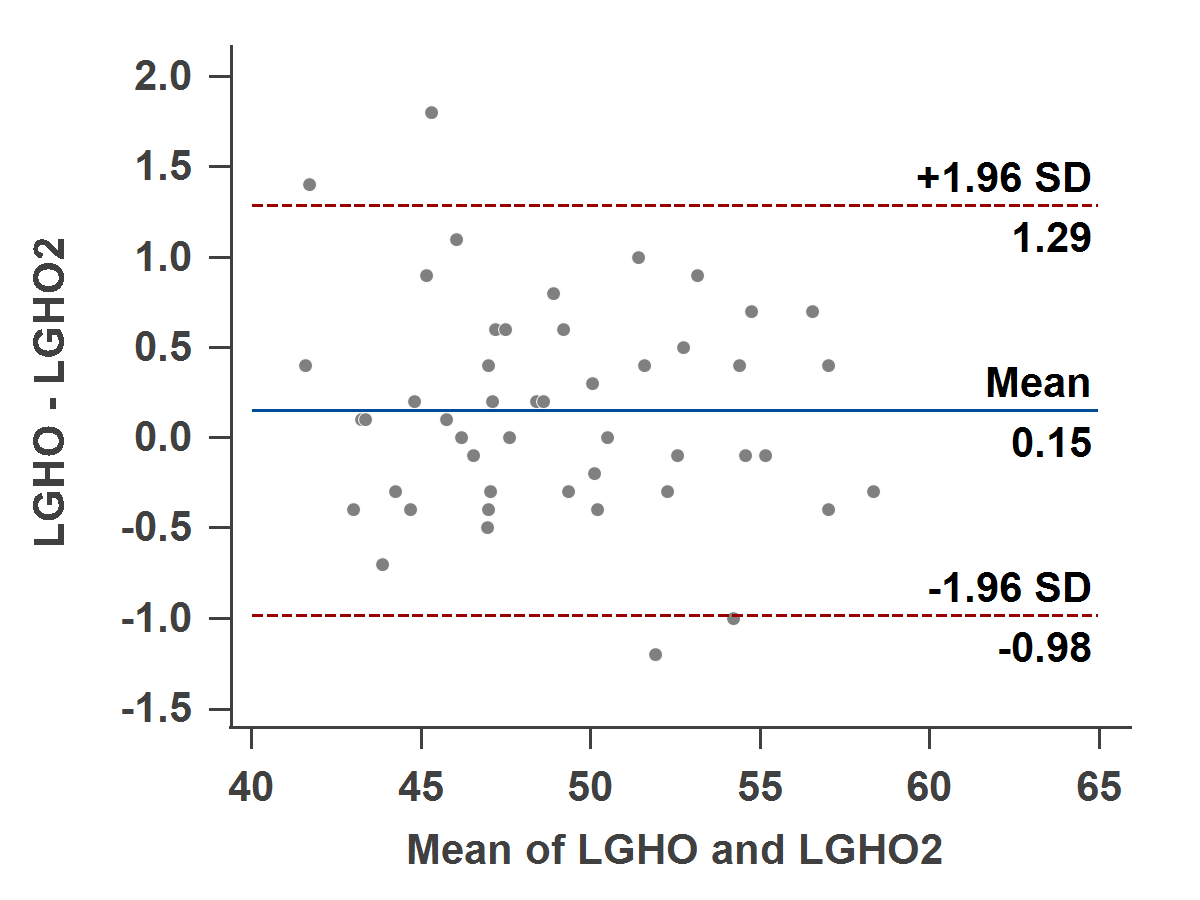

Supplement: Supplementary file 1 [file diagnostics-15-00146-s001.zip › Figure S8_LGHO.tif]

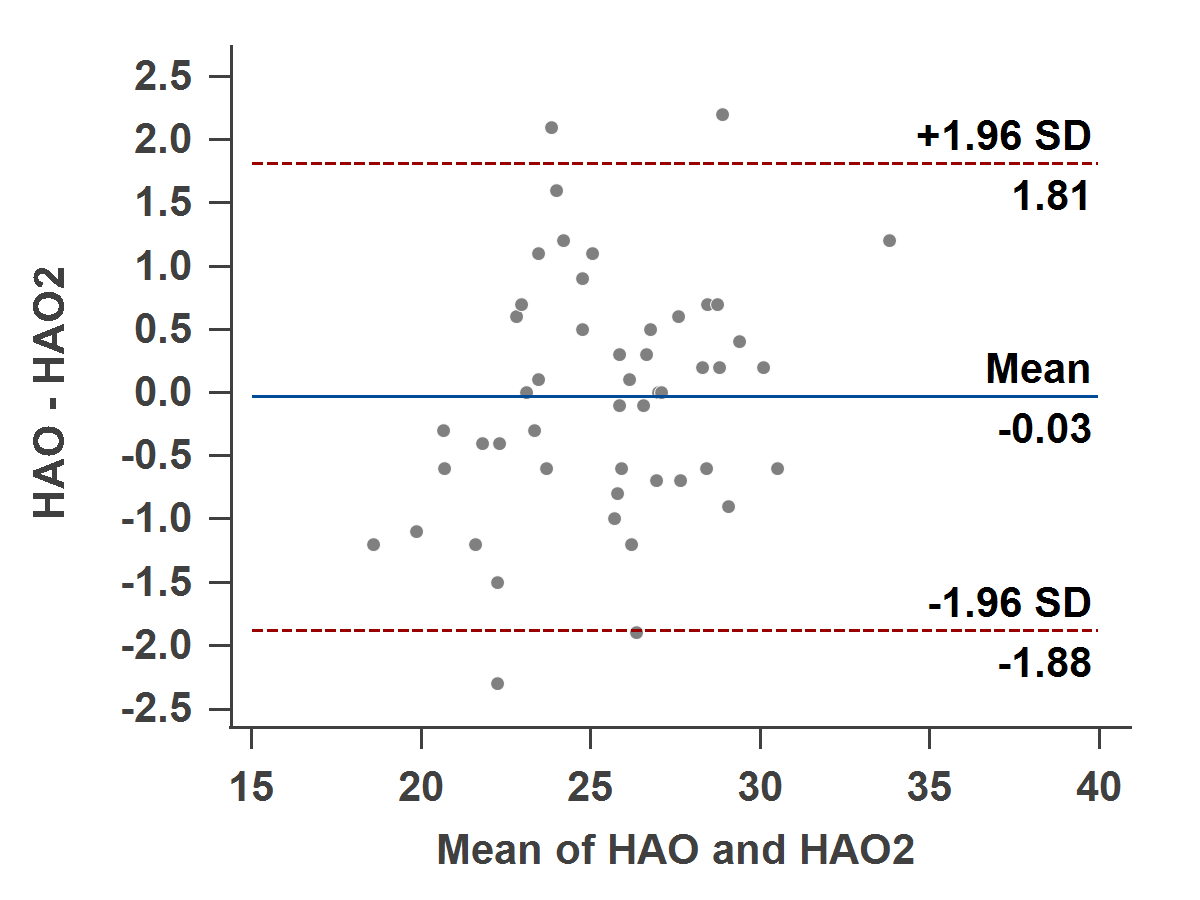

Supplement: Supplementary file 1 [file diagnostics-15-00146-s001.zip › Figure S9_HAO.tif]
